# Sources of Thermal Power Generation and Their Influence on the Operating Temperature of Organic Solar Cells

**DOI:** 10.3390/nano12030420

**Published:** 2022-01-27

**Authors:** Hooman Mehdizadeh-Rad, Kiran Sreedhar Ram, Farhad Mehdizadeh-Rad, David Ompong, Daniel Dodzi Yao Setsoafia, Naveen Kumar Elumalai, Furong Zhu, Jai Singh

**Affiliations:** 1 Energy and Resources Institute and College of Engineering, IT and Environment, Charles Darwin University, Darwin, NT 0909, Australia; hooman.mehdizadehrad@cdu.edu.au (H.M.-R.); kiran.sreedharram@cdu.edu.au (K.S.R.); david.ompong@cdu.edu.au (D.O.); danieldodziyao.setsoafia@cdu.edu.au (D.D.Y.S.); naveenkumar.elumalai@cdu.edu.au (N.K.E.); 2 Department of Electrical and Computer Engineering, University of Texas at Dallas, Dallas, TX 75080, USA; farhadrad@utdallas.edu; 3 Department of Physics and Institute of Advanced Materials, Hong Kong Baptist University, Kowloon Tong, Hong Kong; frzhu@hkbu.edu.hk

**Keywords:** organic solar cell, operating temperature, thermal power, energy off-sets

## Abstract

Thermal stability, closely associated with the operating temperature, is one of the desired properties for practical applications of organic solar cells (OSCs). In this paper, an OSC of the structure of ITO/PEDOT:PSS/P_3_HT:PCBM/ZnO/Ag was fabricated, and its current-voltage (*J*-*V*) characteristics and operating temperature were measured. The operating temperature of the same OSC was simulated using an analytical model, taking into consideration the heat transfer, charge carrier drift-diffusion and different thermal generation processes. The simulated results agreed well with the experimental ones. It was found that the thermalization of charge carriers above the band gap had the highest influence on the operating temperature of the OSCs. The energy off-set at the donor/acceptor interface in the bulk heterojunction (BHJ) was shown to have a negligible impact on the thermal stability of the OSCs. However, the energy off-sets at the electrode/charge-transporting layer and BHJ/charge-transporting layer interfaces had greater impacts on the operating temperature of OSCs at the short circuit current and maximum power point conditions. Our results revealed that a variation over the energy off-set range from 0.1 to 0.9 eV would induce an almost 10-time increase in the corresponding thermal power generation, e.g., from 0.001 to 0.01 W, in the cells operated at the short circuit current condition, contributing to about 16.7% of the total solar power absorbed in the OSC.

## 1. Introduction

**Figure 1:**
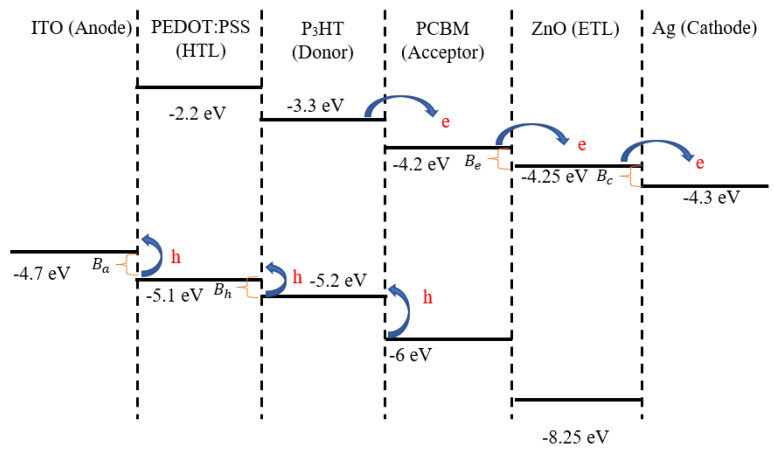
Schematic energy level alignment of functional materials used in the OSC, comprising a layer configuration of ITO/PEDOT:PSS (40 nm)/P_3_HT:PCBM (90 nm)/ZnO (10 nm)/Ag (100 nm).

Organic solar cells (OSCs) have achieved very rapid developments over the last decade, as they are light weight, flexible, and economical to fabricate due to their low temperature solution processing [1,2,3,4,5,6]. The experimental power conversion efficiency (PCE) of OSCs has reached over 18% [7]. To accomplish commercialization, their PCE and stability should be comparable with the commercially available inorganic solar cells [8,9,10,11]. There are various factors that affect the PCE and stability of OSCs, among which the operating temperature plays one of the crucial roles [12,13,14]. The stability and PCE of OSCs degrade due to prolonged operation at high temperatures [15]. Katz et al. [16] have investigated the influence of operating temperature on the performance of polymer-fullerene-based solar cells. They have shown that the open-circuit voltage $\left(V_{oc}\right)$ decreases linearly with increases in the operating temperature. According to Tvingstedt et al. [17], the ideality factor of OSCs is also temperature-dependent, and it provides necessary information about the main charge carrier recombination routes. Lee et al. [13] studied the performance of OSCs at high operating temperatures, ranging from 300 K to 420 K, and used a metal oxide hole-transporting layer to improve the thermal stability of OSCs. Sivula et al. [18] and Bertho et al. [19] investigated the influence of crystallinity of photovoltaic polymers on the thermal stability of OSCs. A photocrosslinkable donor–acceptor conjugated polymer for use in BHJ OSCs, which has shown higher thermal stability, has been developed by Griffini et al. [20]. The importance of glass transition temperature on the thermal stability of polymer solar cells has been studied by Müller [21], and the thermal stability of non-fullerene-based all polymer OSCs has recently been reviewed by Ye et al. [21,22]. Zhang et al. [23] found that small differences in morphology can significantly affect the kinetics and thermodynamic equilibrium of BHJ microstructures, as well as the photostability and thermal stability of the PCE_11_:PCBM solar cells. Lindqvist et al. [24] showed that PC_61_BM and PC_71_BM as an acceptor with a non-crystalline thiophene-quinoxaline copolymer as donor in OSCs can improve the thermal stability. Chen et al. [25] fabricated thermally stable OSCs by incorporating a small amount of a polymer insulator polyacenaphthylene with a high glass-transition temperature over 230 °C into polymer. Zhang et al. [26] fabricated thermally stable OSCs using a small molecule donor with suppressed π-π stacking between molecular backbones which introduced large steric hindrance. Wang et al. [27] showed that the thermal stability of P_3_HT:PC_61_BM blend can be improved by incorporating a porphyrin compound to prevent the PC_61_BM aggregation. Despite the above research on the thermal stability of OSCs, the thermal power generating factors which influence the operating temperature, and subsequently the thermal stability, have not yet been quantitatively well studied. The operating temperature of OSCs can depend on both external and internal factors [28]. The external factors include solar radiation, ambient temperature, wind velocity, sky temperature and surrounding temperature. The internal factors include the heat generation due to the thermalization of charge carriers generated by the absorption of photons of energy higher than the band gap energy, the tail state recombination and transferring the charge carriers through the energy off-sets at the interfaces. In order to understand and quantify different factors that may influence the operating temperature of OSCs, we fabricated and simulated a simple OSC of the structure of indium tin oxide (ITO)/poly(3,4-ethylenedioxythiophene)-polystyrene sulfonate (PEDOT:PSS)/Poly(3-hexylthiophene) (P_3_HT) and phenyl-C61-butyric acid methyl ester (PCBM)/zinc oxide (ZnO)/silver (Ag). Figure 1 shows the energy band diagram for each layer material for this OSC [29,30]. When the electron of an exciton excited in the donor moves to the acceptor, a charge transfer (CT) exciton is created and the excess energy equal to the energy difference between the donor’s Lowest Unoccupied Molecular Orbital (LUMO) and the acceptor’s LUMO is generated in the form of molecular vibrations [31]. Likewise, when the hole of an exciton excited in the acceptor moves from the acceptor’s Highest Occupied Molecular Orbital (HOMO) to the donor’s HOMO, this creates a CT exciton by generating the excess energy in the form of molecular vibrations. These excess energies, if equal or larger than the CT exciton binding energy, impact back to the CT exciton and may dissociate it into free electron hole pairs. We assumed that all the free charge carriers are thus generated through this dissociation mechanism, and all the excess vibrational energy gets used in the dissociation process without influencing the operating temperature. This assumption is justified because in the OSC structure considered here (Figure 1), both energy off-sets of LUMO and HOMO are large enough to dissociate both singlet and triplet excitons. However, after the exciton dissociation, electrons move from acceptor (PCBM) LUMO to the lower energy conduction band of the electron-transporting layer (ETL) (ZnO), and finally to the cathode (Ag) also at the lower energy. Both of these excess energies are lost into the OSC as heat, and are denoted by $B_{e}$ and $B_{c}$, respectively, as shown in Figure 1. Likewise, the free holes, after the exciton dissociation, move from the donor (P_3_HT) HOMO to the HOMO of the PEDOT:PSS and finally to the anode (ITO), and will release the excess energies denoted by $B_{h}$ and $B_{a}$, respectively, (see Figure 1). Thus, after an exciton dissociation in the active layer, when the electron reaches the cathode, an energy equal to $B_{e}$ + $B_{c}$ will be converted to heat, and similarly an energy equal to $B_{h}$ + $B_{a}$ will be converted into heat when the hole reaches the anode.

In this paper, we considered three sources of thermal power generation, thermalization, energy off-sets at the interfaces and tail-states recombination, to simulate the operating temperature of an OSC using the Optical Transfer Matrix Method, and drift-diffusion equations incorporated with the heat transfer mechanisms. The simulated results were compared with the measured operating temperature and J-V characteristics of the above OSC fabricated in our laboratory. The results of this paper could help in the production of stable and more efficient OSCs, by the understanding of the operating temperature-dependent factors and the dependence of operating temperature on the energy off-sets.

## 2. Experiment and Field Test

**Figure 2:**
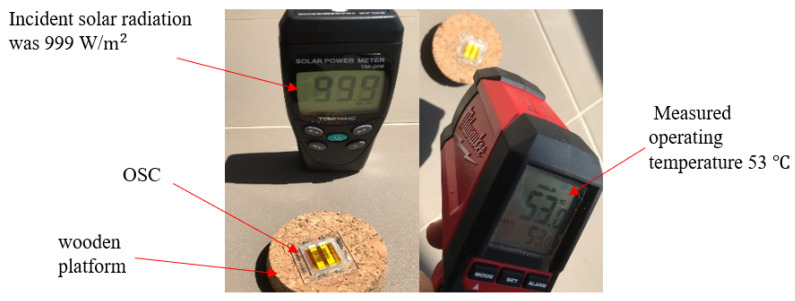
The measured incident solar radiation and operating temperature of the OSC in Darwin.

An OSC with the layer configuration of ITO/PEDOT:PSS (40 nm)/P3HT:PCBM (90 nm)/ZnO (10 nm)/Ag (100 nm), a pre-patterned ITO/glass substrate, with a substrate dimension of 25 × 25 mm^2^ and a sheet resistance of 10 Ω/square, was fabricated for comparison studies. First, the substrate was cleaned by ultra-sonication sequentially with dilute detergent solution, deionized water, acetone, and isopropanol for 30 min each, and then dried using a pure nitrogen stream. For depositing PEDOT:PSS, first, we pipetted it in a vial and put it in the ultrasonic machine for 10 min to become uniform. For a better deposition, we placed the substrate in the UV plasma machine for 10 min before depositing PEDOT:PSS. The PEDOT:PSS was first filtered, and then pipetted on the substrate by a syringe for spin coating with a rotation speed of 5000 rpm for 30 s, which deposited a thickness of about 40 nm. We mixed 500 μL orthodichlorobenzene (ODCB), 12.5 mg P3HT and 10 mg PCBM (1:0.8), and the solution thus prepared was placed on a hot plate (50 °C) with the magnetic stirring bar for 24 h. Next, using a spin coater, we deposited 40 μL of the organic active layer blend of P3HT: PCBM with a rotation speed of 2000 rpm for 30 s, which deposited a thickness of 90 nm. In the next step, with the same technique, 30 μL ZnO was deposited on the cleaned ITO substrate with a rotation speed of 2500 rpm for 15 s, which deposited ZnO of a thickness of about 10 nm. Finally, we deposited 100 nm Ag by a thermal evaporator; thus, fabricated OSC was encapsulated. The operating temperature of the fabricated OSC shown in Figure 2 was measured with a thermometer to be 53 °C, along with the incident solar radiation of 999 W/m^2^ was measured using a solar radiation meter under the open circuit voltage (unload) condition in Darwin, a tropical city in Australia, in October 2020. As the measured incident solar radiation was very close to the standard 1000 W/m^2^ at AM 1.5G, this field test was carried out very close to the standard condition. In the above experiment, OSC was placed on a wooden platform, which had a very low thermal conductivity, the wind velocity was measured to be 2 m/s, and the ambient temperature was 306 K. As organic solar cells are very thin, their Biot number is much less than 0.1 according to lumped capacitance method. Therefore, we could assume that the temperatures of the surface of the cell and that of the cell were the same and the temperature gradient within the thin film solar cells was negligible. This has been discussed in our earlier work [32] for thin film perovskite solar cells.

## 3. Methodology

The ground methodology is based on our previous works [14,28,32] on simulating the operating temperature of a perovskite solar cell, where we incorporated the influence of other factors such as grain boundary sizes and tale state recombination rates at the interfaces and grain boundaries. As this paper focuses on OSCs, and organic solids have different properties than perovskites, it is necessary to outline the theoretical details here again, without repetition. Following our earlier work [14,28,32], we have assumed that the OSC operates under the steady state condition, i.e., ∂T/∂t=0, where T is the operating temperature, and t is time. Thus, for an illuminated OSC shown in Figure 2, we have solved the energy balance equation given by:

(1) $$Ir\alpha A-P_{G}+P_{Rec}+P_{B}=h_{c,amb}A\left(T-T_{amb}\right)+h_{r}A\left(T-T_{amb}\right)$$

where Ir is the incident solar radiation ($W/m^{2}$), α is absorbance, A is the solar cell area ($m^{2})$ and $P_{G}\;$is the absorbed solar power to generate the photo-excited electron in LUMO and hole in HOMO (W), and it can be written as [28]:

(2) $$P_{G}=qGE_{g}Ad$$

where G is the rate of total electron-hole pair generation (s^−1^m^−3^), $E_{g}$ is band gap energy and d is active layer thickness (m).

**Figure 3:**
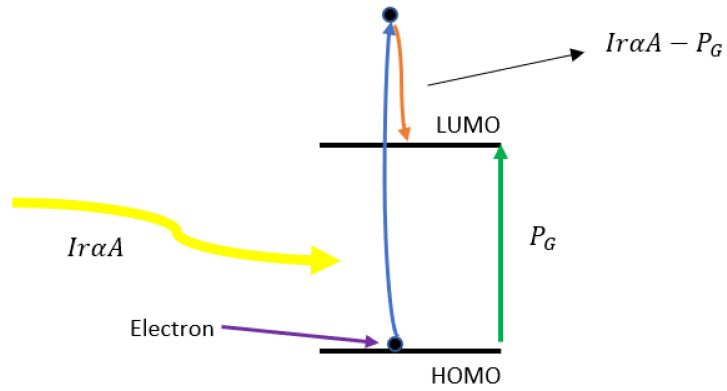
The schematic diagram of heat generation due to thermalization. An electron from HOMO is excited to higher energy than LUMO and then relaxes to LUMO by releasing the excess power IrαA.

When a photon of energy greater than the band gap is absorbed, it can excite an electron from HUMO to a higher energy beyond LUMO; then, it relaxes down to LUMO by releasing the excess energy as thermal energy, which is referred to as thermalization and the associated thermal power thus generated is equal to $Ir\alpha A-P_{G}$ as shown in Figure 3.

Some of the electrons and holes generated due to the absorbed solar power, $P_{G}$, may recombine non-radiatively and generate the thermal power denoted by $P_{Rec},$ and some may generate thermal power $P_{B}$ by moving to lower energy due to energy off-sets while transferring towards their respective electrodes. Thus,$\;P_{Rec}$, the total thermal power generated due to the non-radiative recombination of the photo-generated electron and hole pairs, can be expressed as $P_{Rec}$ [28]:

(3) $$P_{Rec}=P_{Rec-GB}+P_{Rec-Int}+P_{Rec-Other},$$

where$\;P_{Rec-GB}$, $P_{Rec-Int}$ and$\;P_{Rec-Other}$ are the thermal powers generated at the grain boundaries (GBs), interfaces (Int) and other parts (Other), respectively, in the active layer. The thermal power generated due to the tail state recombination at the GBs can be given by [28]:

(4) $$P_{Rec-GB}=qR_{tail-GB}E_{g}V_{GB},$$

where $R_{tail-GB}$ (s^−1^m^−3^) is the average tail state recombination rate per unit volume, and $V_{GB}$ is the total volume of GBs (m^3^). The schematic geometry of GBs is assumed to be spherical of diameter $d_{GB},$ distributed (m) in the whole active layer (the details are presented in our previous work [28]). The thermal power generated due to the tail state recombination at the interfaces $P_{Rec-Int}$ can be written as [28]:

(5) $$P_{Rec-Int}=qR_{tail-Int}E_{g}V_{Int},$$

Where $R_{tail-Int}$ (s^−1^m^−3^) is the average tail state recombination rate at per unit volume of the interfaces,$\;V_{Int}=2A_{Int}d_{Int}\;$is the volume of the two interfaces at either end of the active layer, $A_{Int}$ and $d_{Int}$ are the area and depth of each interface, respectively. In this simulation $d_{Int}=2\;$nm is assumed to be the thickness of each interface within which the tale state recombination may occur. Both thicknesses, $d_{GB}\;$and $d_{Int}$, are assumed to be the same for simplifying the simulation.

The thermal power generated due to the tail state recombination at other parts in the active layer $P_{Rec-Other}\;$can be determined by [28]:

(6) $$P_{Rec-Other}=qR_{tail-Other}E_{g}V_{Other},$$

where $R_{tail-Other}$ (s^−1^m^−3^) is the average tail state recombination rate per unit volume in other parts of the active layer, and $V_{Other}$ is given by:

(7) $$V_{Other}=V_{AL}-V_{GB}-V_{Int}.$$

where $V_{AL}\;$is the volume of the active layer.

$P_{B}$ is the thermal power generated due to the transport of free charge carriers to their respective electrodes through the energy off-sets, and can be given by:

(8) $$P_{B}=q\left(G-R\right)BAd,$$

where R is the total recombination rate including radiative (Langevin recombination [33,34]) and non-radiative recombination (tail state recombination [34,35]), and B is the total energy off-set, and can be written as (see Figure 1):

(9) $$B=B_{h}+B_{a}+B_{e}+B_{c}.$$

We assumed that the sky and the surroundings had the same temperature as ambient temperature. Therefore, the radiation heat transfer coefficient from the solar cell to the sky and the surrounding area can be determined by [14,28]:

(10) $$h_{r}=\varepsilon_{c}\sigma_{sb}\left(T+T_{amb}\right)\left(T^{2}+T_{amb}{}^{2}\right)$$

where $\varepsilon_{c}$ is the emissivity coefficient of solar cell, $\sigma_{sb}=5.67\times 10^{-8}$ (W$m^{-2}K^{-4})\;$is the Stefan–Boltzmann constant.

$h_{c,c-amb}$ (W$m^{-2}K^{-1})\;$in Equation 1 is the convection of the heat transfer coefficient from the solar cell to the ambient, and can be determined by the empirical equation [36,37]:

(11) $$h_{c,c-amb}=5.62+3.9\;U$$

where U is the wind velocity, and the numbers 5.62 and 3.9 are extracted empirically from the experiments.

We calculated G using the Optical Transfer Matrix Method. *R,* the total of the radiative and non-radiative recombination rates, was calculated using drift-diffusion equations in which the heat transfer equations were incorporated [28,38]. Then, we used Equations (1)–(11) to simulate the operating temperature of the OSC. The details of the procedure of simulation of the operating temperature are presented in our previous work [14,28,32].

## 4. Results and Discussion

**Figure 4:**
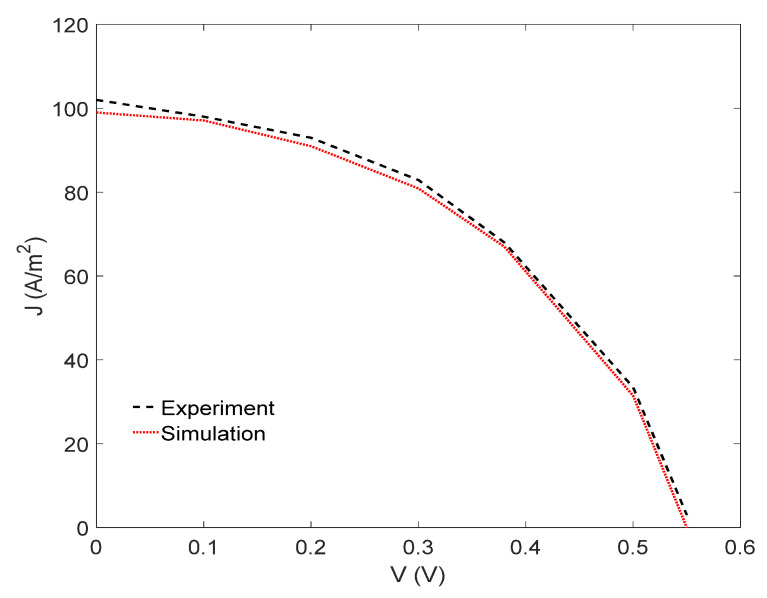
Comparison between the measured and simulated J-V characteristics of the OSC of the structure: ITO/PEDOT:PSS (40 nm)/P3HT:PCBM (90nm)/ZnO (10 nm)/Ag (100 nm).

To validate our simulation, first we calculated and measured the J-V characteristics of the fabricated OSC, as shown in Figure 4. According to Figure 3, our simulation J-V curve agreed very well with the experimental ones. The input data that we used in the simulation are listed in Table 1.

**Figure 5:**
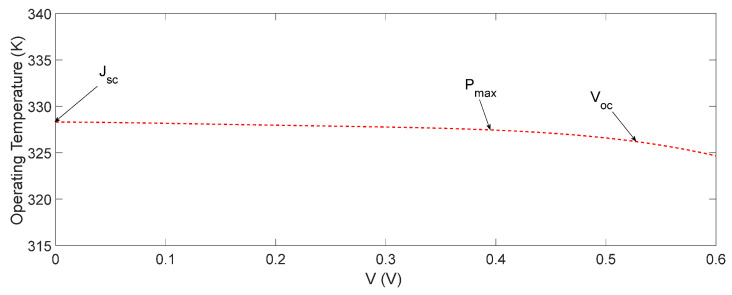
The simulated operating temperature of the OSC as a function of voltage. $J_{sc\;}$, $P_{max}$ and $V_{oc\;}$ marked by arrows.

Next, we simulated the operating temperature using the process described in the previous section and plotted it in Figure 5 as a function of the voltage. According to Figure 5, the operating temperature of the OSC decreased gradually by increasing the voltage, and it was about 326.8 K at the $V_{oc\;}$ condition, which was consistent with our experimental result, shown in Figure 2, of 326 K.

**Figure 6:**
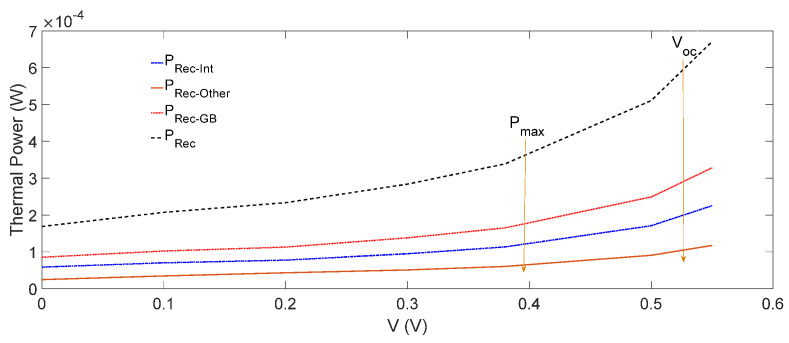
The thermal power generated due to the non-radiative recombination at GBs$\;\left(P_{Rec-GB}\right)$, interfaces ($P_{Rec-Int}$ ), and other parts of the active layer $\left(P_{Rec-Other}\right)$ as a function of the voltage across the cell and the total thermal power generated due to the non-radiative recombination$\;P_{Rec},\;$ is shown by the black-dashed curve.

**Figure 7:**
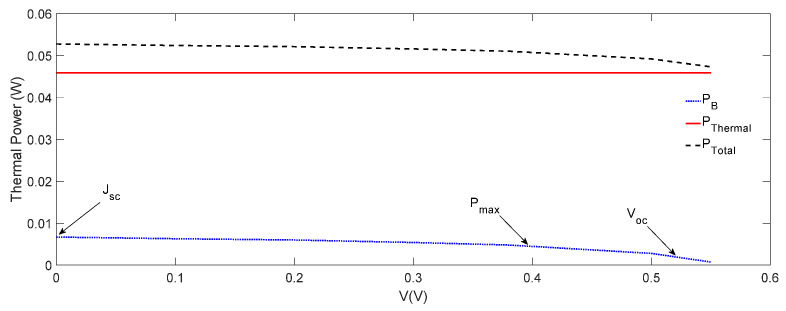
Total thermal energy-generated $P_{B\;}$due to the transfer of free charge carriers to the respective electrodes through the energy off-sets $B_{e},\;B_{c},\;B_{h}\;\mathrm{and}\;B_{a}$; thermal power-generated $P_{Thermal}$ due to thermalization of charge carriers above the band gap and the total thermal power-generated $P_{total}$ =$\;P_{Thermal}$ + $P_{B}$ + $P_{Rec}$ as a function of voltage.

In order to understand the influence of different thermal power components on the operating temperature of OSCs, we proceeded as follows. We calculated and plotted the thermal powers $P_{Rec-GB}$, $P_{Rec-Other}$ and $P_{Rec-Int}$ in Figure 6 and $P_{Thermal}$ and $P_{B}$ in Figure 7 as a function of the voltage. As it is shown in Figure 6, by increasing the voltage, $P_{Rec-GB}$, $P_{Rec-Other}$ and $P_{Rec-Int}$ increased, and this increase became more pronounced at higher voltages. This was expected, as by increasing the voltage closer to $V_{oc\;}$ (no current), the non-radiative recombination rate increased, because less charge carriers were collected by the electrodes. Therefore,$\;P_{Rec}$, which is the total thermal power generated due to the non-radiative recombination of the photo-generated electron and hole pairs (Equation (3)), was about $1.7\times 10^{-4}$ W at $J_{sc\;},\;$and $4.2\times 10^{-4}$ W at $V_{oc\;}\;$condition, which is relatively very small. On the other hand, at a higher applied voltage, as less charge carriers were collected at the electrodes, the the thermal power generated, $P_{B}$ due to the energy off-sets will reduce.

We plotted both $P_{B}$ and $P_{Thermal}$ as a function of voltage in Figure 7, where $P_{Thermal}$ = IrαA− $P_{G}=$ 0.044 W, and was independent of the voltage, and $P_{B}=0.006$ W at $J_{sc\;}$ decreased slightly by increasing the voltage. These values of $P_{B}$ and $P_{Thermal}$ in Figure 7 are relatively much higher than the total thermal power$\;P_{Rec}$ generated through the non-radiative recombination in Figure 6. However, as $P_{B}$ was of the order of $10^{-3}\;$W in comparison wth $\;P_{Rec}$ $\approx 10^{-4\;}W$, the former played the dominant role. Therefore, as $P_{B}$ decreased slightly, but $P_{Thermal}$ remained constant with the increase in voltage (Figure 7), the small decrease in the operating temperature with the voltage (Figure 5) could be attributed to the decrease in $P_{B}$. It may be re-emphasized that in calculating $P_{B}$, we assumed that the donor–acceptor energy off-sets were converted to the vibrational energy required for the dissociation of CT excitons, and hence would not influence the operating temperature of the OSCs, as described above. It may also be noted that $P_{B}$, $\;P_{Rec}$ and $P_{Thermal}$ depended on the energy off-sets within the structure and the materials used in different layers, and hence their magnitude may have varied from one OSC to another. For the OSC considered in this paper, $P_{B}$ was relatively significant. If, however, the energy off-sets are reduced by interface engineering, then $P_{B}$ can be minimised, and one will receive a different operating temperature dependence on the voltage than that obtained in Figure 5.

At the $J_{sc\;}$condition, most of the photoexcited holes and electrons are transported to their respective electrodes, and hence there is a minimum recombination rate in the active layer. Thus, $P_{Rec}$ will be the minimum at the $J_{sc\;}$condition, but $P_{B}$, the thermal power generated by the energy off-sets, will be the maximum, because all charge carriers go through the energy off-sets in the structure of the OSC. As mentioned above, at the $V_{oc\;}$condition, there was no current flow in OSC, and hence all photoexcited charge carriers were accumulated in the active layer, and recombined radiatively or non-radiatively; only the non-radiative recombination contributes to thermal power, while the radiative recombination to light. As a result, at the $V_{oc\;}$condition, $P_{Rec}$ becomes the maximum, and $P_{B}$ the minimum, because there is no transport of charge carriers through the energy off-sets. Thus, at the $J_{sc\;}$condition, $P_{Rec}$ is the minimum, and $P_{B}$ the maximum, and at the $V_{oc\;}$condition $P_{Rec}$ is maximum and $P_{B}$ the minimum. Hence, $P_{Rec}+{\;P}_{B}$ contributing to the operating temperature will not be the same at the $J_{sc\;}$and $V_{oc\;}$conditions. As explained above, for the OSC considered here, $P_{B}$ is more than $P_{Rec}$ and plays the dominant role. Therefore, $P_{Rec}+{\;P}_{B}$ decreases by increasing the voltage, and subsequently the operating temperature also decreases by increasing the voltage (see Figure 5), which means that the operating temperature at the $J_{sc\;}$condition is the highest and lowest at the $V_{oc\;}$condition. At the maximum power point, $P_{Rec}+{\;P}_{B}$ will be lower than that at the $J_{sc\;}$condition, and higher than that at the $V_{oc\;}$condition, as clearly shown in Figure 5.

We also calculated the total thermal power generated using $\;P_{total}\;$=$\;P_{Thermal}\;$+$\;P_{B}\;$+$\;P_{Rec}$, and the results are shown in Figure 7. As it is shown in Figure 7, $\;P_{total}$ decreases slightly by increasing the voltage due to a slight decrease in$\;P_{B}$, which is consistent with the operating temperature shown in Figure 5.

The values of various thermal powers and the operating temperature at $J_{sc\;}$, $V_{oc\;}$, and $P_{max}$ of the OSC: ITO/PEDOT: PSS (40 nm)/P3HT: PCBM (90 nm)/ZnO (10 nm)/Ag (100 nm) with the energy off-set B = 0.6 eV are listed in Table 2, along with the associated standard deviation. As it can be seen in Table 2, $P_{Thermal}\;$had the highest contribution in the operating temperature of this OSC. $P_{B}$ was the second most important factor at $J_{sc\;}$and $P_{max}$, but it becomes zero at $V_{oc\;}$.

**Figure 8:**
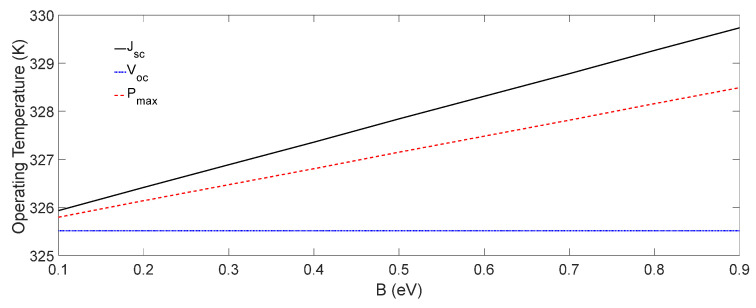
The operating temperature of OSCs as a function of total energy off-set B at $J_{sc\;}$, $V_{oc\;}$, and $P_{max}$ conditions.

**Figure 9:**
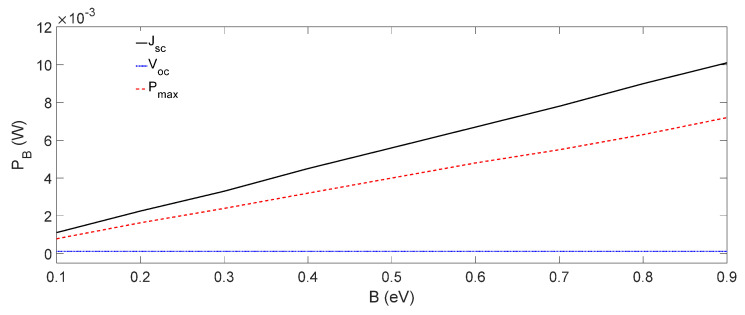
$P_{B}$ plotted as function of B at $J_{sc\;}$, $V_{oc\;}$, and $P_{max}$ conditions.

We also investigated the influence of the total energy off-sets B on the operating temperature of the OSC. As it is shown in Figure 8, the operating temperature increased linearly with B at $J_{sc\;}$and $P_{max}$ conditions, and remained constant at the $V_{oc\;}\;$condition. It may also be noted that the slope of the operating temperature, with respect to B at the $J_{sc\;}$condition, was larger than that at the $P_{max}$ condition, and this implies that the dependency of the operating temperature of OSCs on the energy off-set was more at the $J_{sc\;}$condition. This is because at the $J_{sc\;}$condition, more electrons and holes were transferred to the electrodes through the energy off-sets,; therefore, $P_{B}$ increases. However, at the $V_{oc\;}$condition, the charge carriers were not transferred to the electrodes, and hence $P_{B}\;$becomes negligible, as shown in Figure 9.

In order to investigate the influence of B on the operating temperature of OSCs further, $P_{B}$ was calculated at $J_{sc\;}$, $V_{oc\;}$, and $P_{max}$ conditions, and the results are shown in Figure 9. As it can be seen in Figure 9, similar to the operating temperature (Figure 8), $P_{B}$ also increased linearly with B, having different slopes at the $J_{sc\;}$and $P_{max}$ conditions and a constant at the $V_{oc\;}$condition. According to Figure 9, it may also be noted that as B increased from 0.1 eV to 0.9 eV, $P_{B}$ increased 10 times, from about 0.001 to 0.01 W at the $J_{sc\;}$condition, which is 16.7% of the total power IrαA = 0.06 W absorbed in the solar cell (see Table 2).

## 5. Conclusions

We solved the heat transfer and drift-diffusion equations to simulate the operating temperature of an organic solar cell by incorporating all the thermal power-generating components listed in Table 2. The simulated operating temperature and *J*-*V* characteristics of the organic solar cell considered in this work were validated by comparing with the corresponding experimental results. The results show that among all the internal thermal power-generating factors, the thermalization of charge carriers above the band gap had the highest influence on the thermal stability and operating temperature of the organic solar cell. It was shown that the acceptor–donor energy off-sets had no significant influence on the operating temperature of an organic solar cell. However, the operating temperature varied linearly with the sum of the anode, cathode, hole and electron transport layer energy off-sets at both a short circuit current and the maximum power point conditions, but it remained constant at the open circuit voltage condition. It was found that if the total energy off-set B increased from 0.1 eV to 0.9 eV, the corresponding thermal power $P_{B}$ generated increased almost 10 times from about 0.001 W to 0.01 W at the short circuit current condition, which is about 16.7% of the total solar power IrαA = 0.06 W absorbed in the solar cell.

## Figures and Tables

**Table 1:** List of input parameters used for simulation in this paper.

Parameter	Value
Ir $\left({\mathrm{wm}}^{-2}\right)$ Solar irradiance	1000 (Measured)
U (m/s) Wind (or air) velocity	1 (Lab condition)
$T_{a}$ (K) Ambient temperature	300 (Lab condition)
α Absorbance	0.6 (Fitting Parameter)
$E_{g}$ (eV) Bandgap	1 [38]
d (nm) Active layer thickness	90 (Measured)
$d_{GB}$ Grain boundary diameter (nm)	100 (Fitting Parameter)
$\mu_{n}$ Mobility of electrons $\left(m^{2}V^{-1}s^{-1}\right)$	$3.5\times 10^{-8}$ [38]
$\mu_{p}$ Mobility of holes $\left(m^{2}V^{-1}s^{-1}\right)$	$10^{-9}$ [38]
$N_{tInt}\;$Density of tail states at interface (${\mathrm{cm}}^{-3}{\left(\mathrm{eV}\right)}^{-1}$)	$10^{17\;}$(Fitting Parameter)
$N_{tGB}\;$Density of tail states at GB (${\mathrm{cm}}^{-3}{\left(\mathrm{eV}\right)}^{-1}$)	$10^{17\;}$(Fitting Parameter)
$N_{tO}$ Density of tail states at other positions of the active layer $({\mathrm{cm}}^{-3}{\left(\mathrm{eV}\right)}^{-1}$)	$10^{16\;}$(Fitting Parameter)

**Table 2:** Simulated values of each factor which influence the operating temperature of the OSC at $J_{sc}$, $V_{oc\;}$, and $P_{max}$ conditions.

Parameters	$At\;J_{sc\;}\mathrm{Condition}\;$	$At\;V_{oc\;}\;\mathrm{Condition}$	$At\;P_{max}\;\mathrm{Condition}$	Standard Deviation
T (K)	328.3	326.8	327.6	0.750555
$P_{Rec-GB}$ (W)	$8.5\times 10^{-5}$	$2.3\times 10^{-4}$	$1.2\times 10^{-4}$	$7.57\times 10^{-5}$
$P_{Rec-Int}$ (W)	$5.8\times 10^{-5}$	$1.2\times 10^{-4}$	$8.1\times 10^{-5}$	$3.13\times 10^{-5}$
$P_{Rec-Other}$ (W)	$2.4\times 10^{-5}$	$0.7\times 10^{-4}$	$4.0\times 10^{-5}$	$2.34\times 10^{-5}$
IrαA (W)	0.060	0.060	0.060	0
$P_{G}$ (W)	0.016	0.016	0.016	0
$P_{Thermal}$ (W)	0.045	0.045	0.045	0
$P_{B}$ (W)	0.006	≈0	0.004	$3.05\times 10^{-3}$

## Data Availability

The data presented in this study are available in this article.
